# An Approach toward Automatic Specifics Diagnosis of Breast Cancer Based on an Immunohistochemical Image

**DOI:** 10.3390/jimaging9010012

**Published:** 2023-01-04

**Authors:** Oleh Berezsky, Oleh Pitsun, Grygoriy Melnyk, Tamara Datsko, Ivan Izonin, Bohdan Derysh

**Affiliations:** 1 Department of Computer Engineering, West Ukrainian National University, Lviviska, 11, 46003 Ternopil, Ukraine; 2 Department of Pathological Anatomy with Section Course and Forensic Medicine, I. Horbachevsky Ternopil National Medical University, 1 Maidan Voli, 46001 Ternopil, Ukraine; 3 Department of Artificial Intelligence, Lviv Polytechnic National University, 79013 Lviv, Ukraine

**Keywords:** immunohistochemical images, diagnosis, image preprocessing, segmentation

## Abstract

The paper explored the problem of automatic diagnosis based on immunohistochemical image analysis. The issue of automated diagnosis is a preliminary and advisory statement for a diagnostician. The authors studied breast cancer histological and immunohistochemical images using the following biomarkers progesterone, estrogen, oncoprotein, and a cell proliferation biomarker. The authors developed a breast cancer diagnosis method based on immunohistochemical image analysis. The proposed method consists of algorithms for image preprocessing, segmentation, and the determination of informative indicators (relative area and intensity of cells) and an algorithm for determining the molecular genetic breast cancer subtype. An adaptive algorithm for image preprocessing was developed to improve the quality of the images. It includes median filtering and image brightness equalization techniques. In addition, the authors developed a software module part of the HIAMS software package based on the Java programming language and the OpenCV computer vision library. Four molecular genetic breast cancer subtypes could be identified using this solution: subtype Luminal A, subtype Luminal B, subtype HER2/neu amplified, and basalt-like subtype. The developed algorithm for the quantitative characteristics of the immunohistochemical images showed sufficient accuracy in determining the cancer subtype “Luminal A”. It was experimentally established that the relative area of the nuclei of cells covered with biomarkers of progesterone, estrogen, and oncoprotein was more than 85%. The given approach allows for automating and accelerating the process of diagnosis. Developed algorithms for calculating the quantitative characteristics of cells on immunohistochemical images can increase the accuracy of diagnosis.

## 1. Introduction

According to the American Statistical Register, in 2020, there were approximately 2.3 million new breast cancer cases and 685,000 breast cancer death cases worldwide. Breast cancer incidence and mortality varied among countries, with age-standardized incidence ranging from the highest of 112.3 per 100,000 population in Belgium to the lowest of 80 per 100,000 population in Iran, and the age-standardized mortality was from the highest of 41.0 per 100,000 population on the island of Fiji to the lowest of 6.4 per 100,000 population in South Korea.

The disease analysis that has been conducted since 2000 revealed that the peak age of breast cancer in some countries in Asia and Africa was ten years earlier than in Europe or America. Regarding breast cancer tendencies, the age-standardized incidence rates increased significantly in China and South Korea and decreased in the United States between 2000 and 2012. Meanwhile, the age-standardized mortality rates increased substantially in China and South Korea but decreased in the United Kingdom, the United States, and Australia between 2000 and 2015. Analyzing the data, we can conclude that regardless of the region of residence, the problem of cancer is relevant and requires tools for diagnosis.

Immunohistochemistry is one of the effective tools for breast cancer preoperative diagnosis.

Immunohistochemistry (IHC) is used to find specific protein products, observe the unique characteristics of breast cancer, differentiate breast cancer from cancer in other locations, obtain information about genetic changes, study prognostic factors, and provide their effective treatment. Technical developments allow for the use of IHC as a comfortable diagnostic tool and its application in advanced protocols in combination with other histochemical methods. Standardization and quality control are critical issues for the acceptable use of IHC in breast cancer practice.

The American Society of Clinical Oncology and the College of American Pathologists convened an international expert group that conducted a systematic review and evaluation of the literature in partnership with Cancer Care Ontario and developed recommendations for optimal ER/PgR test efficiency. The Commission recommends determining the status of ER and PgR for all invasive breast cancers and recurrent breast cancers. It is recommended that ER and PgR tests be considered positive if the sample contains at least 1% positive tumor nuclei during testing in the typical reactivity of internal (usual epithelial elements) and external controls.

Biomedical images cannot be archived. Therefore, the image is uploaded into memory in its original format. The next stage is preliminary processing including input parameter identification, adaptive filtering, and brightness/contrast adjusting.

Images obtained from a microscope are characterized by noise and the absence of precise contours of cell nuclei. Therefore, it is necessary to develop an algorithm for image preprocessing. The ultimate goal of the preprocessing stage is to remove impulse noise and align the histogram.

Thus, the results of the immunohistochemical study are immunohistochemical images. Immunohistochemical images are processed using artificial intelligence for diagnosis.

Artificial intelligence is widely used in medicine. These can be both software (stationary and mobile) and hardware modules and devices, which allows for the speeding up and automation of diagnostics. The relevance of breast cancer research and the use of AI for these tasks are explored in the research study [1].

With hardware development, new software tools appeared that use immunohistochemical images to make a diagnosis. Scientists have also paid great attention to the development of software that combines algorithms for image processing, segmentation, object detection, etc. Analysis of the latest publications in this area is provided in Section 2.

Modern software complexes such as ImageJ are characterized by a large number of tools for the manual or semi-automatic image processing of experimental samples. The disadvantages of this system are the need for computer vision knowledge and time for complex and monotonous work. In addition, some programs have functionality that allows highlighting only areas with cell nuclei. Therefore, diagnosticians need to independently calculate the cell nuclei parameters. We offer a software package that allows for automatic calculation of the micro-objects’ quantitative characteristics in immunohistochemical images. To do this, the program uses a knowledge base for preprocessing and segmentation algorithms. The result of the program is the identification of the disease subtype. Taking into account the current trends in machine learning, in future research, it is planned to use neural networks with the U-net architecture for automatic image segmentation, which will allow for more accurate identification of micro-objects in the image.

The main contribution of this paper can be summarized as follows:

- We developed an algorithm for image preprocessing that was based on adaptive median filtering with experimental determination of the image noise level, and identification of the filter window size, which allowed for a reduction in the impulse noise level on the input image;
- We proposed a combined segmentation algorithm based on the watershed and threshold segmentation algorithms to calculate the area and identify the cell staining intensity. It will allow for the determination of informative indicators for breast cancer subtype identification;
- We developed a method of the automatic statement of specified diagnosis based on the preliminary processing algorithms and histological and immunohistochemical image segmentation using brightness indicators and relative area. This made it possible to determine the breast cancer subtype automatically;
- We developed a software module within the HIAMS software system, implemented in the Java programming language using the OpenCV computer vision library.

The article consists of the following structural parts: (1) Introduction: the relevance of the immunohistochemical study of breast cancer is shown. (2) Literature analysis: publications on automatic diagnosis are analyzed. (3) Materials and methods: the method of diagnosis based on the image immunohistochemical analysis is described. (4) Results, comparisons, discussions: the developed module of automatic diagnosis and network data is described, and the developed system’s comparative analysis with known ones is carried out. (5) Conclusions: our conclusions are presented.

## 2. Literature Review

Let us overview the artificial intelligence tools to analyze immunohistochemical images and automated diagnosis.

In [2], the authors investigated the possibility of automated breast cancer diagnosis. Immunohistochemical (IHC) images, image segmentation algorithms, and neural network methods were analyzed in [3,4]. An automatic breast cancer diagnosis was performed using textural features, entropy, and classifiers SVM, CNN, and DTree. These methods of automation and data analysis also require time and hardware. However, their efficiency and speed are better than manual diagnostics based on biomarkers [5,6,7,8,9]. There are several problems with the accuracy and reliability of the dataset, data gaps, noise, anomalies, etc. The use of biomarkers in digital image analysis was considered in [10].

The significant contribution of scientists to the development of algorithms for the automatic selection and calculation of cell nuclei parameters emphasizes the importance of this problem. The use of biomarkers allows for the highlighting of necessary nuclei in the image. However, the absence of clear contours, touching and crossing of nuclei in the immunohistochemical image causes the development of preprocessing and segmentation algorithms.

Existing means of artificial intelligence do not provide sufficient prediction accuracy in diagnosis. The use of deep learning methods to classify many classes of pathologies based on image analysis was studied in [11,12,13]. CNN architecture optimization using bio-inspired algorithms for breast cancer detection was considered in [14].

In [15], the authors investigated the segmentation and classification of the IHC image nuclei using biomarkers. The authors used two semi-automatic software: NuclearQuant v. 1.13 and Pannoramic Viewer v. 1.13. 1.14. Each of these programs determines the status of biomarkers on the analyzed micro-object.

Software tools for breast cancer diagnosis were discussed in [16]. The authors defined a system of diagnosis and prognosis of the disease, Diaprog, which used the data of the classified and archived care records. In this study, a significant emphasis was placed on data processing, however, the algorithm for calculating the quantitative cell nuclei characteristics and making a diagnosis based on them is not given.

The article in [17] was devoted to the software tool for analyzing IHC images for the quantitative assessment of tissue pathology. The authors developed an automated IHC_Tool procedure with TIFF images at a magnification of _200 to quantify the cell traits. Automatic classification of cancer cells using machine learning was analyzed in [18]. The method of automated machine learning for differentiation of the invasion method was formed in the article.

In [19], the authors described the use of a Faster R-CNN object detector with four function extractors: Resnet-50, VGG-16, Inception-V2, and Resnet-101 for automatic lymphocyte detection and counting. In [20], the authors presented an automatic computer-aided diagnosis system based on the Multimodal fusion of Breast Cancer (MF-CAD).

In [21], the researchers investigated new methods of segmentation and calculation of the IHC image cancer cell nuclei. These methods segment nuclei based on modified superpixel segmentation. In [22], IHC methods for the use of immunotherapy in oncology were explored. The authors described various highly multiplexed methods that allow for the simultaneous detection of multiple markers on a single tissue section. The authors in [23] discussed the methods of analysis of IHC images based on deep learning using CNN and U-Net. However, only a few studies have focused on automatic diagnosis using many biomarkers.

Thus, these articles provided the impetus to develop algorithms and software systems to speed up and improve the quality of the diagnosing process. However, these programs do not provide a diagnosis based on the immunohistochemical images of different types of biomarkers. In addition, there is a need to develop universal segmentation algorithms for different types of images.

The authors in [24] analyzed classical and new approaches for automatic diagnosis in oncology using machine learning. A broad analysis of classical and in-depth teaching methods used in the histological image analysis of images was presented. In articles [25,26], the technique of image analysis based on the pathologist-tree network was investigated, and an automatic system of analysis of the IHC images was developed. In addition, an automated rapid visualization system using a synchronized 12-LED illuminator was developed.

Feasibility in the quantification of Ki-67, ER, PR, and HER2 biomarkers was proven in [27]. However, the article did not provide algorithms and software tools for diagnosis based on the analyzed biomarkers.

In [28,29], the approach to automatic biomedical image segmentation using U-Net convolutional neural network technology is presented. However, in this case, taking into account the specifics of immunohistochemical images, a different approach to segmentation was chosen. An adaptive method of biomedical image segmentation based on metrics was developed in [29]. This approach uses the rules based on segmentation algorithms. In [30,31,32,33,34,35], strategies to analyze biomedical images based on data processing algorithms are presented. The research findings demonstrate approaches to the development of adaptive methods of cytological and histological image preprocessing and segmentation based on fuzzy logic. However, there is a need to develop alternative methods of immunohistochemical processing and the automatic identification of the cancer subtype based on the obtained indicators.

## 3. Materials and Methods

We used the following symbols to describe the method of the diagnostic statement:

— Pr is progesterone;
— *Er* is estrogen;
— *HER2/neu* is the oncoprotein;
— *Ki-67* is the cell proliferation biomarker;
*S_w_* is the area of a field of view window;
— *S_p_* is the area of positive cells in the field of view;
— *δ_s_* is the ratio of the area of positive cells in the field of view to the area of the field of view window;
— *KI* is the color intensity coefficient;
— G is the degree of tumor differentiation based on the histological image analysis;
— $BC_{A}$ is the subtype Luminal A of breast cancer (BC);
— $BC_{B}$ is the BC subtype Luminal B;
— $BC_{H}$ is the BC amplified subtype;
— $BC_{Z}$ is the BC subtype basaltic;
— $ER_{\sigma_{S}}$ is the relative area of the cell nuclei (estrogen biomarker);
— $PR_{\sigma_{S}}$ is the relative area of the cell nuclei (progesterone biomarker);
— $HER2_{\sigma_{S}}$ is the relative area of the cell nuclei (biomarker oncoprotein);
— $KI67_{\sigma_{S}}$ is the relative area of the cell nuclei (a biomarker of cell proliferation);
— $ER_{K_{I}}$ is the level of color intensity of the cell nuclei (biomarker estrogen);
— $KI67_{KI}$ is the level of color intensity of the cell nuclei (a biomarker of cell proliferation);
— *T_L_* is the lower segmentation threshold (thresholding);
— *T_H_* is the upper segmentation threshold (thresholding).

### 3.1. Method of Diagnostic Statement Based on Immunohistochemical Image Analysis

To make a diagnosis, we used the histological images IG. As a result of the action of biomarkers such as progesterone, estrogen, oncoprotein, and the cell proliferation biomarker, for each histological image, we obtained four immunohistochemical images: $I_{C_{1}},I_{C_{2}},I_{C_{3}},I_{C_{4}}$. Thus, the input is the following set of images:

$$I=\left\{I_{G},I_{C_{1}},I_{C_{2}},I_{C_{3}},I_{C_{4}}\right\}.$$

To make an accurate specified diagnosis, we analyzed the immunohistochemical images in two leading indicators, $\delta_{S}$ and $K_{I}$.

The method of the specified diagnosis consists of the following steps:

*Image preprocessing*.

Each of these images is a microscopic image with pulsed noise. Therefore, it is necessary to preprocess the images. To do this, we calculated the peak signal-to-noise ratio (PSNR).

Let *I* be the input image (histological or immunohistochemical image). As a result of median filtering over the input image, we obtain:

$$I^{I}=M\left(I\right)$$

where *I* is the input image and $I^{I}$ is the result of the median filtering.

The next step is to quantify the noise level of the image. We used the value of the peak signal-to-noise ratio (PSNR) [36].

We calculated the standard deviation (MSE) between the two images to calculate this value.

$$MSE=\frac{1}{mn}\;\overset{m-1}{\underset{i=0}{\sum }}\;\overset{n-1}{\underset{j=0}{\sum }}{|I^{I}\left(i,j\right)-I^{\;}\left(i,j\right)|}^{2},$$

where $I^{I}$ and I are the filtered original images, respectively, size *m* × *n*. The value of PSNR is determined as follows:

$$PSNR=10log_{10}(\frac{MAX_{I}^{2}}{MSE}),$$

where $MAX_{I}$ is the maximum value accepted by the pixel of the image. Experimental studies have established the following parameters of the median filter window:

$$\left\{\begin{array}{l} mw=5\times 5,\;\;\;PSNR\leq 20\;dB\\ mw=3\times 3,\;\;\;PSNR>20\;dB \end{array}\right.$$

where *mw* is the size of the median filter window.

To reduce the level of impulse noise, we used a median filter with a window size of *mw*. We present the image filtering as follows:

$$I^{II}=mw\times I^{I},$$

where $I^{I}$ is the input image; mw is the filter window; $I^{II}$ is the image after filtering.

To calculate the average brightness of the image, we used the following transformation:

$$Y=\frac{1}{n}\sum_{i=0}^{n}0.299\times R_{i}+0.587\times G_{i}+0.114\times B_{i},$$

where *n* is the total number of pixels in the image; *R_i_*, *G_i_*, *B_i_* are the values of red, green, and blue channels; and the th pixel of the image, respectively [37].

The following parameters *α* were selected experimentally depending on *Y*’s average brightness level. Using the *α* parameter, we adjusted the image brightness. Therefore, this image was better processed during the segmentation. Selection of the parameter was carried out by experimental selection of the value of α with step 2 and further analysis of the obtained image after segmentation.

$$\alpha =\left\{\begin{array}{c} 20;\;Y\leq 10\\ 12;\;10<Y\leq 40\\ 8;\;40<Y\leq 150\\ 6;150<Y\leq 200\\ 4;\;Y>200 \end{array}\right.$$

Based on the defined parameter *α*, we performed the following image transformation

$$I^{III}=\alpha \times I^{II}$$

### 3.2. Segmentation and Calculation of Cell Staining Intensity Area

At this stage, we segmented the obtained images to determine the cell staining intensity area. We chose a combination of a watershed algorithm based on markers and a threshold. As a result of the algorithm use, we obtained a mask with a segmented image, where the pixels of one segment were marked with the same label and formed a connected area. The main disadvantage of this algorithm is the use of a pre-processing procedure for images with a large number of local minima. The standard watershed algorithm of the OpenCV library was selected as the basis of the algorithm, which includes elements of threshold segmentation, erode and dilate operations, and the generation of markers. The main emphasis was placed on the choice of the lower and upper thresholds.

Each image type is unique. Therefore, the lower (*T_L_*) and upper (*T_H_*) segmentation thresholds for a specific image type were experimentally selected:

$$\left\{\begin{array}{l} T_{L}=160,T_{H}=180;\mathrm{Pr}\\ T_{L}=180,T_{H}=210;Er\\ T_{L}=40,T_{H}=230;HER2/neu\\ T_{L}=160,T_{H}=180;Ki-67 \end{array}\right.$$

We present the segmentation by the watershed method as follows:

$$I^{IV}=\alpha \times I^{III}$$

The next step is to calculate the ratio of the positive cell area in the field of view to the area of the field of the view window.

$$\delta_{S}=\frac{S_{p}}{S_{w}},0\leq \delta_{S}\leq 1.$$

To calculate the intensity of cells, we imposed the segmented image on the input image and calculated the intensity of the selected areas.

The image intensity *K_i_* was calculated according to the scale from 1 to 3. In usual practice, diagnosticians describe the intensity with the words “high”, “medium”, and “low” based on their own subjective experience. To develop an automatic system, we converted qualitative characteristics into quantitative ones. The sources of information were the image samples and descriptions made by diagnosticians.

$$\left\{\begin{array}{l} 0\leq Y\leq 15;K_{i}=1\\ 16\leq Y\leq 30;K_{i}=2\\ Y>30;K_{i}=3 \end{array}\right.$$

### 3.3. Determination of Breast Cancer Molecular Genetic Subtype

The degree of tumor differentiation G is divided into three types:

- $G_{1}$—a highly differentiated tumor;
- $G_{2}$—a moderately differentiated tumor;
- $G_{3}$—a low differentiated tumor.

The molecular genetic subtype of breast cancer is determined based on four biomarkers.

***Luminal subtype A*** is characterized for $G_{1}$, and $G_{2}$ is defined by the following system of features:

$$\left.BC_{A}=\left\{\begin{array}{l} ER_{\sigma_{S}}>0.66,ER_{K_{I}}=3,\\ PR_{\sigma_{S}}>0.2,\\ HER2_{\sigma_{S}}<0.1,KI67_{\sigma_{S}}<0.2 \end{array}\right.\right|$$

***Luminal subtype B*** is characterized for $G_{2}$, and $G_{3}$ is defined by the following system of features:

$$\left.BC_{B}=\left\{\begin{array}{l} ER_{\sigma_{S}}<0.66,ER_{K_{I}}=3,\\ PR_{\sigma_{S}}<0.2,\\ HER2_{\sigma_{S}}<0.1,K_{I}=1,KI-67_{\sigma_{S}}>0.2 \end{array}\right.\right|$$

The amplified subtype *HER2/neu* is characterized for $G_{3}$ and defined by the following system of features:

$$\left.BC_{H}=\left\{HER2_{\sigma_{S}}<0.1,K_{I}=1,ER_{\sigma_{S}}<0.1,PR_{\sigma_{S}}<0.1\right.\right|$$

The following system of features defines the basal-like subtype:

$$\left.BC_{Z}=\left\{\begin{array}{l} ER_{\sigma_{S}}<0.1,PR_{\sigma_{S}}<0.1,\\ HER2_{\sigma_{S}}<0.1,KI-67_{KI}=3 \end{array}\right.\right|$$

## 4. Results, Comparison, and Discussion

### 4.1. Dataset Description

For the computer experiments, the immunohistochemical image dataset of breast cancer was used [38]. Table 1 shows the parameters of the selected dataset.

The sample for automatic segmentation was divided into training and tests in a 60 to 40 percent ratio.

### 4.2. Software Module Structure

**Figure 1:**
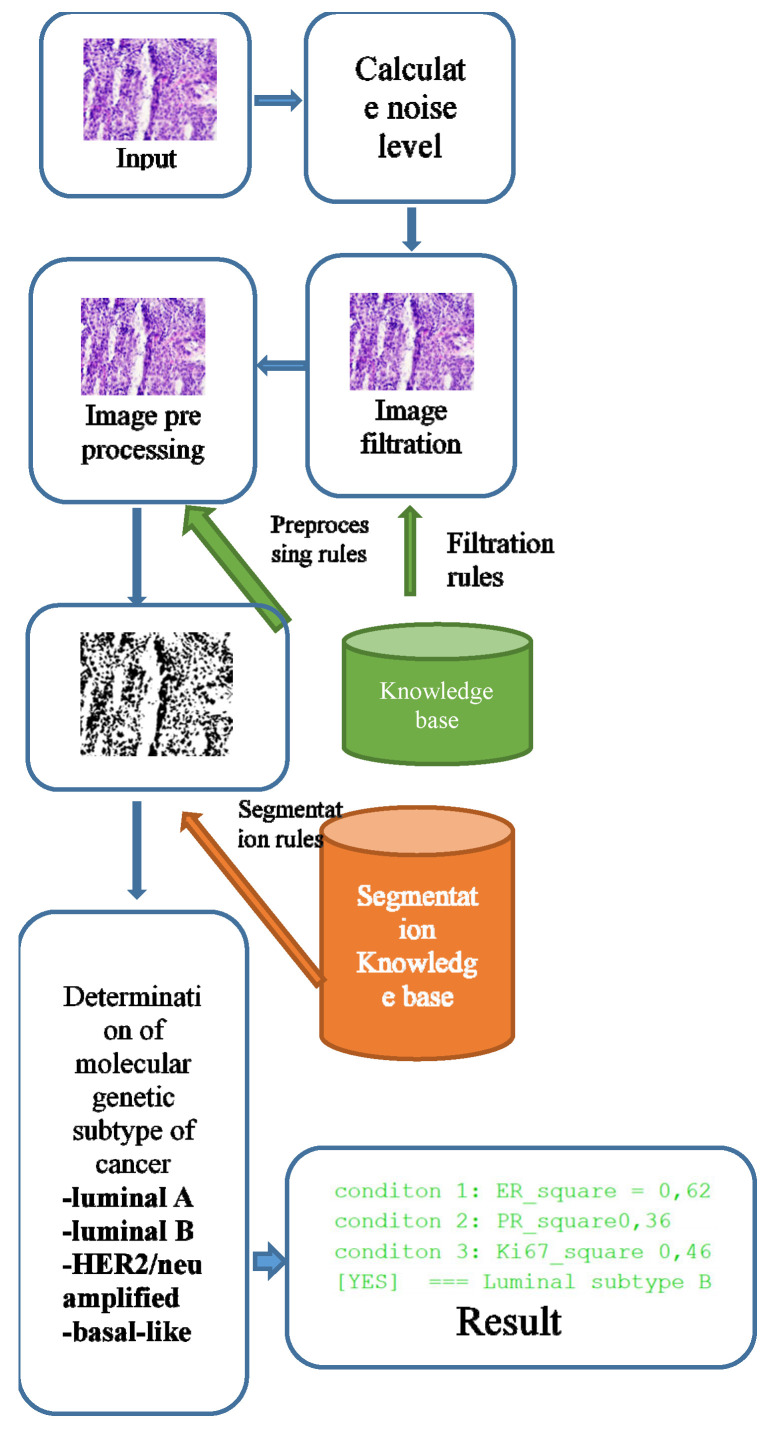
Software module of the generalized structure.

The software module was implemented using the Java programming language and the openCV library. Figure 1 shows a generalized structure.

The knowledge base for the selection of filtering and segmentation algorithms was implemented in the form of “IF–THEN” type rules. An example of the rules for selecting the parameters of the filtering algorithms and the brightness level adjustment parameters is given in Section 3.1. The filter algorithm was selected according to the following rules:

**IF** the peak signal-to-noise ratio <= 20, **THEN** the median filter window = 5 × 5;
**IF** the peak signal-to-noise ratio >20, **THEN** the median filter window = 3 × 3;

The rules for choosing the parameters of the segmentation algorithms are given in Section 3.2. The rules in “IF_THEN” format are as follows:

**IF** Image Type = progesterone **THEN** thresholds lower = 160 AND thresholds upper = 180;
**IF** Image Type = estrogen **THEN** thresholds lower = 180 AND thresholds upper = 210;
**IF** Image Type = oncoprotein **THEN** thresholds lower = 40 AND thresholds upper = 230;
**IF** Image Type = cell proliferation biomarker **THEN** thresholds lower = 160 AND thresholds upper = 180.

The input parameters were the following image characteristics: noise level, peak signal-to-noise ratio, and image type (Her2/neu, Ki-67, Er, Pr). A filtering or segmentation algorithm and its parameters were provided for each range of input values. Training was carried out by testing images with predefined parameters. This stage is computationally complex and requires parallelization. The best results were recorded into the knowledge base. When a new image is received, the parameters of the algorithms are automatically selected depending on the selected image input values.

A stack of immunohistochemical and histological images enters the software module. First, we calculated the noise level. A median filtering was performed based on the noise level with a window size corresponding to the parameter selection rules.

Images obtained as a result of microscopic examination were characterized by uneven illumination, obscuration areas, or, conversely, excessive illumination. An image preprocessing unit was used to adjust the brightness level. Brightness adjustment was based on the rules stored in the knowledge base.

It is necessary to determine the relative area of the cell nuclei in the image and the average level of brightness of the cell nuclei to make a diagnosis. To do this, it is necessary to segment the images and superimpose the segmented image on the original to calculate the brightness level.

After the segmentation stage, the brightness level is calculated. The next step is to calculate the cancer subtype’s conditions directly.

Class “Main” is an entry point to the software module. This class provides methods for determining the image path and storing intermediate versions of the image after performing certain operations such as filtering. ArrayList is used to store image parameters that are used for further processing. The fields “ER_”, “PR_”, “HERN2_”, “KI67” are designed to store information about the parameters that are used to determine a particular cancer subtype.
1String imagePath = myMap.get(0).get(pathKey);2System.***out***.println(pathKey + **“= >”** + imagePath);3Mat originalMat = **new** Mat();4originalMat = Highgui.*imread*(imagePath);5StartImageParams stip = **new** StartImageParams();6stip.getStartValues(originalMat);7Teaching teaching = **new** Teaching(originalMat, imagePath);8teaching.generateImages(stip, originalMat,160,180);9System.***out***.println(**“relative area”** + teaching.getSquarePercent() +10**“ =-----= Average brightness level”** + teaching.getGetHistogramAverage());11Ki kis = **new** Ki(teaching.getGetHistogramAverage());12*Kintensiive* = kis.getStatus();

An example of a code for determining the relative area and level of brightness is as follows:
1**private boolean** condition_1(){2**int** ER_square_new = (**int**) (**this**.ER_square*100 + 10);3**if**(ER_square_new > 66 && **this**.**ER_intense_status** == 3.0) {4System.*out*.println(**“\033[0;32m condition 1”** + **“\033[0m”**);5}**else** {6System.*out*.println(**“\033[0;31m condition 1”** + **“\033[0m”**);7}8 9 10**if**(ER_square_new > 66 && **this**.**ER_intense_status** == 3.0){11**return true**;12}13 14**return false**;15}

This class was designed for automatic immunohistochemical and cytological image segmentation. The class consisted of a constructor, additional methods, and fields for storing an image object in the OpenCv Mat format.

### 4.3. Computer Experiments

**Figure 2:**
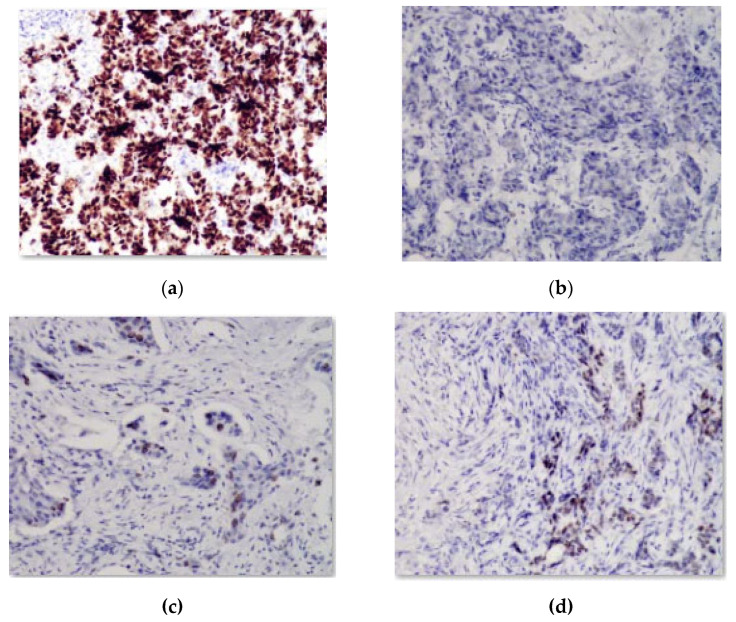
Image with reaction to (**a**) estrogen; (**b**) oncoprotein, (**c**) cell proliferation biomarker; (**d**) progesterone.

A software module was developed for computer experiments, which automatically defines a preliminary diagnosis. The software module is a part of the HIAMS software package [39]. Examples of immunohistochemical images are shown in Figure 2.

These images were obtained from the database of a private immunohistochemical laboratory. The image database was closed.

**Figure 3:**
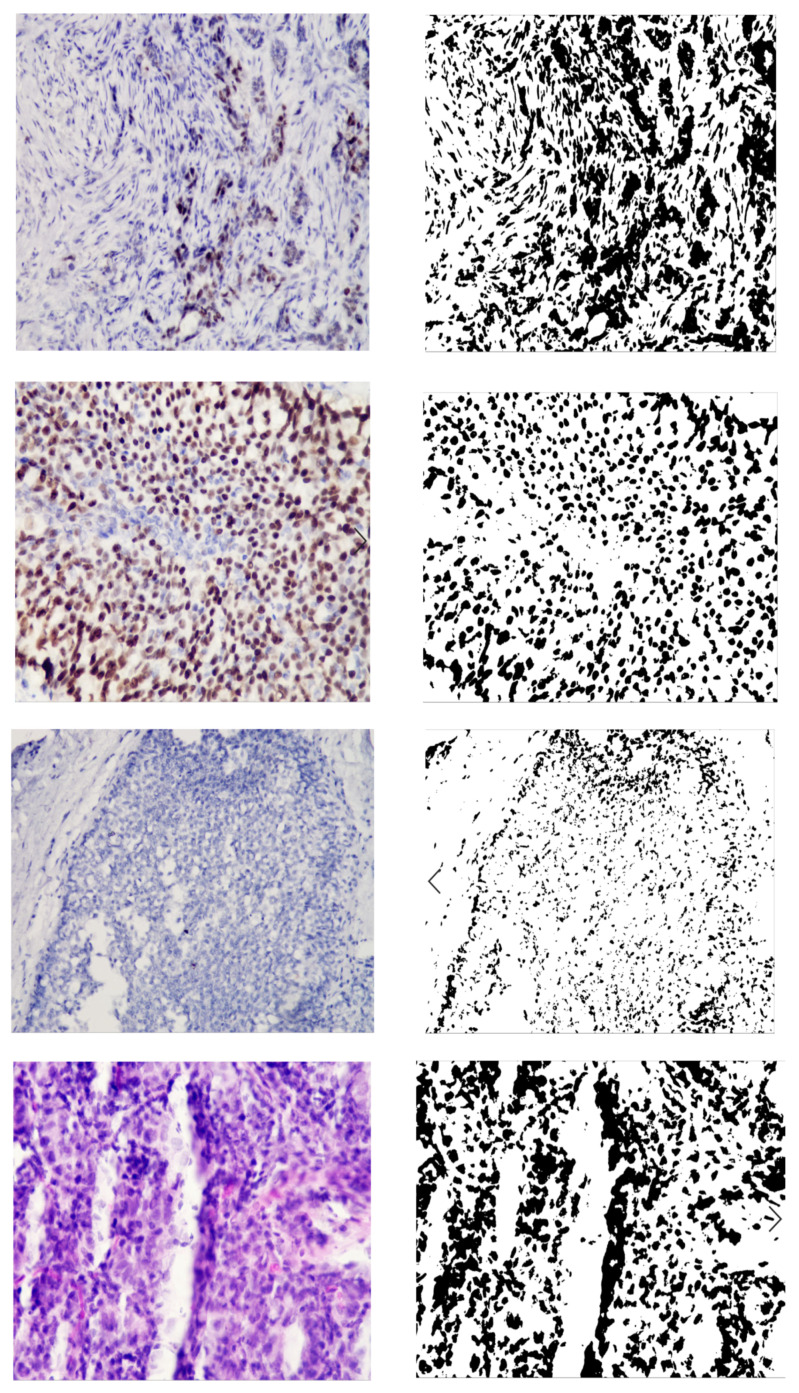
Original and generated images.

**Figure 4:**
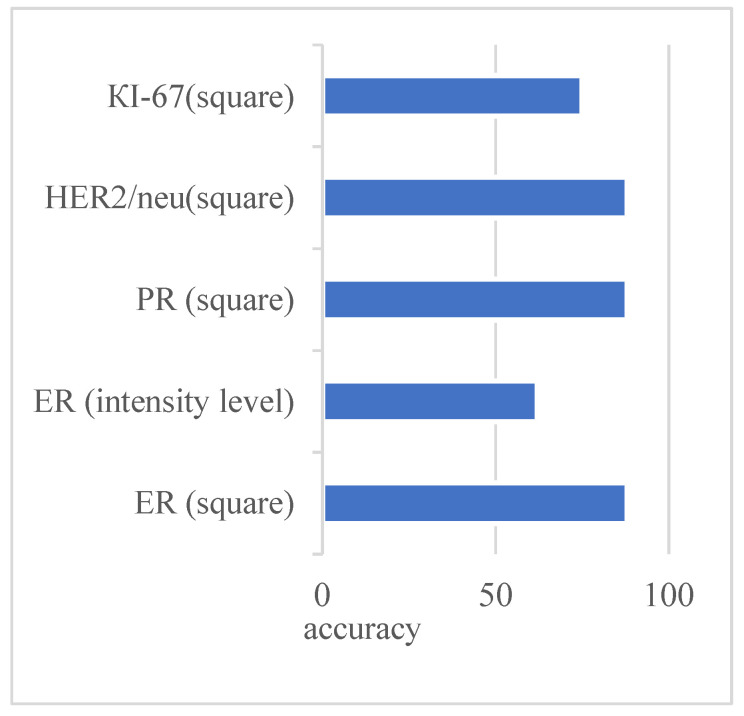
Indicators of the correctly detected parameters for Luminal A subtype.

Figure 3 shows the original image and the generated mask, reflecting the areas of interest. The image is the mask generated automatically without human intervention based on the developed adaptive algorithm of image preprocessing. As can be seen from Figure 3, the studied images were not of the same type. With automatic diagnosing, many calculations were performed on several images. Relative area and cell intensity were used to assess the accuracy of the developed method of preliminary diagnosing. Figure 4 shows the ratio of the correctly defined parameters (area, intensity) to the total number of images in the study. To determine the subtype “Luminal A”, one of the conditions was to calculate the area of cells in the image processed with the biomarker “progesterone”.

Several parameters and conditions characterize each molecular genetic subtype of cancer. Figure 4 shows the number of correctly defined parameters for the subtype Luminal A based on the experimental studies.

The figure shows that the parameters were determined at almost the same level. Only the intensity parameter for the ER image had a low result—62%.

### 4.4. Comparison of Results of Automated Microscopy Systems

Table 2 shows a comparative analysis of some of the automated microscopy systems and developed systems. The indicators for comparison are the following: segmentation algorithms, automatic calculation of quantitative characteristics, automatic calculation of brightness and area, storage in a database, and diagnosis according to the Nottingham scale.

Thus, most automated microscopy systems have a set of algorithms that allow for the calculation of the characteristics of the cell nuclei. However, unlike the developed system, analogs do not have the functionality for automatic diagnosis. Diagnosing in the known automated microscopy systems is only possible in manual or automated mode.

## 5. Conclusions

Advances in artificial intelligence have greatly influenced the development of modern medicine. The visualization of processes in diagnosing various organs makes it possible to identify pathological processes in the early stages. Processing of images obtained during visualization is an urgent and complex problem. A cancer diagnosis is based on cytological, histological, and immunohistochemical image analysis. The use of immunohistochemical images, obtained under the influence of biomarkers, allows for accurate diagnoses. We present a new method of the specified diagnosis of breast cancer subtypes. Pre-processing improved the quality of the input immunohistochemical images. The segmentation was performed based on the watershed and threshold segmentation algorithms. The authors developed the algorithm for determining the molecular genetic subtype of breast cancer: “Luminal A”, “Luminal B”, HER2/neu amplified, and basal-like. Experiments to determine the subtype of breast cancer “Luminal A” based on the calculation of the area and intensity of cells in the image showed high accuracy (more than 80%). Only the intensity parameter for ER showed a result of 62%.

In further studies, the initial histological images should be automatically classified. In addition, in limited initial samples, it is necessary to artificially generate immunohistological images to test the developed algorithms for automatic diagnosis.

## Figures and Tables

**Table 1:** Characteristics of an immunohistochemical image dataset.

Image Class	Number of Images	The Ratio of Sides of One Image	Size of One Image
Estrogen	20	4096 × 3286 pixels	10 Mb
Progesterone	20	4096 × 3286 pixels	10 Mb
Oncoprotein	20	4096 × 3286 pixels	10 Mb
Cell proliferation biomarker	20	4096 × 3286 pixels	10 Mb

**Table 2:** Comparative analysis of automated microscopy systems (“+”—criterion is present, “−”—criterion is absent, “+/−”—criterion is implemented in automated mode).

Parameters	Developed Module	HIAMS	ImageJ	Axio Vision	BioImageXD
Segmentation algorithms The k-means method Watershed Smart scissors	+ + −	+ + −	+ + +/−	+ + +	+ + +
Automatic calculation of quantitative characteristics	+	+	+	+/−	+/−
Automatic detection of brightness and relative area	+	−	−	+/−	+/−
Storage of calculation results in the database	+	+/−	−	+	+
Diagnosis according to the Nottingham scale	+	−	−	−	−

## Data Availability

The datasets generated during and/or analyzed during the current study are available in this paper. The data presented in this study are available on request from the corresponding author. The data are not publicly available due to restrictions of privacy.
